# The First Molar

**Published:** 1892-12

**Authors:** W. B. Conner


ARTICLE II.

### THE FIRST MOLAR.

If the first molar is extracted before the second molar has erupted, we find the space closing up sooner, and to all appearances there has not been any retrograde movement on the anterior teeth. But upon examination of the bite and jaws, we find a change and a change not entirely at the expense of the jaw posterior to the lesion.

It has been taught in our colleges and journals that we must expect the second molar to occupy the space left vacant, and our natural conclusion has been in harmony with these teachings.

It is contrary to all scientific reasoning to expect such a result where extraction takes place at an age when the jaws are so yielding to the forces brought to bear upon them.

How often do we find a first molar moving forward to occupy the space left vacant after extracting the second bicuspid?

I have never observed one such instance. Is it not just as lame an argument to advance as that of a second molar moving forward to occupy the first molar's space?

The models I have passed around are casts of my own mouth showing the arrangement of teeth and changes taken place. You will readily see the absence of the first molars in both upper and lower jaws. The upper were extracted at about the tenth or eleventh year, and the lower were left in the jaw until exfoliated, which was completed at the fifteenth or sixteenth year. So the upper were lost before and the latter several years after the eruption of the second molars, bringing about the change presented.

We have in this case a decided shortening of the bite which is shown by the position of the occluding teeth, instead of an interlocking of the canines and bicuspids which was the normal occlusion prior to the extraction, the superior canines and incisors are striking directly upon the incisive surfaces of the eight anterior lower teeth, proving conclusively a determined shortening of the bite. There is also a loss of the use of six bicuspids, the four superior and two inferior. I might say here that in all cases the lower jaw is not interfered with to near the extent of the upper after extraction, owing no doubt to the fact that the inferior maxillary bone is ossified earlier in life and is of a stronger mould and texture offering more resistance.

I maintain that the superior maxillary in this case has been shortened to the extent of the width of the missing tooth extracted, not saying anything about the constriction in the width of the jaw.

The second inferior molar having erupted earlier than the superior, which is true in the majority of cases, gives it more prestage, having a firmer union with the jaw controlling the movement of its antagonist. The result you observe holding the superior second molar in nearly its normal position, thereby prohibiting an interior movement, but not a rotary movement. The forces brought to bear at this early age upon the superior teeth anterior to the first molar might be regretted as insignificant.

We observe in our every day practice that the protrusions of the superior or inferior canines if let alone, will in the course of a short time be adjusted to the arch and antagonistic teeth. The only force brought to bear is the weight of and contractions of the obicularis oris muscle, with the assistance of the muscles of expression and mastication. If the bones and process is so yielding at the time of the eruption of the canines, why should not the jaw be more so during the eruption of the second molars two years previous ?

There is no doubt that if such changes as above mentioned come about at the age of twelve that the jaws have not become fully developed, and adhering to the law governing repair, the destruction is always the greatest in the direction of the least resistance. We have a retrograde movement in every case of early extraction of the sixth year molar. In the superior maxillary the molar eminence adds its marked features to the face, its consequent absorption, its dire effect upon the stronger expression. In every case of early extraction there is an entire loss of this protuberance, and the muscles and surfaces over it become shortened and flattened with constriction of the face in its vicinity. There is a depression at the alæ-nasi, and a sunken condition of the bones which occasionally extends to the floor of the orbit. In the face of all the abnormalities produced we have our journals full of this or that theory relative to the time when this tooth should be extracted. One writer claims the eight year, another the tenth, and another the eleventh or twelfth, and all for what purpose ? To bring about a better arrangement and condition of the tenth and for the benefit of humanity in general.

I am safe in making the assertion that nature has not produced as many irregularities as the early extraction of the six year molar has occasioned. Does extracting this tooth at an early age preclude all possibility of an over-crowded condition ? Can we foretell the size, development and exact arrangement of the teeth and jaws far into the future ? If such prophetic knowledge is possessed by a few, the whole profession should know it. Nature's plan of eruption is a most harmonious one. When the first molar is erupted it acts as an anchor or foundation to the arch, always in the proper place competently adapted for the purpose intended, and in the course of time the second molar erupts, reinforcing as it were, in accordance with the eternal fitness of things.

Now, are we as a scientific profession going to improve upon nature's plan by removing the foundation and dictate our plan of constructing a human jaw ? We should have not only one object in view from extracting, giving room for incoming teeth and temporary relief. The best safeguard a dentist can have is to preserve if possible all of the sixth year molars until that condition has come about when the bones of the face have become perfectly developed and features set, the second molars, bicuspids and canines held in position by firm occlusion. Then and not until then do I consider it prudent for the first permanent molar to be removed. Everything is conjecture and visionary when we extract this tooth at an early age with such delusions as some authors advocate, to lead us. Our observation if closely applied will not direct or advise such a procedure. The intelligence of the age demands of us all that our knowledge can avail, and it does not show that we are advancing when we resort to such practice as we have been taught to follow in the past.—CONNER, (W. B.,) *Ohio Journal.*

---

ARTICLE III.

### APPLICATION OF THE RUBBER DAM.

